# The Role of MicroRNAs in Myocardial Infarction: From Molecular Mechanism to Clinical Application

**DOI:** 10.3390/ijms18040745

**Published:** 2017-03-31

**Authors:** Teng Sun, Yan-Han Dong, Wei Du, Chun-Ying Shi, Kun Wang, Muhammad-Akram Tariq, Jian-Xun Wang, Pei-Feng Li

**Affiliations:** Institute for Translational Medicine, Qingdao University, Qingdao 266021, China; tengsun@qdu.edu.cn (T.S.); dongyanhande@163.com (Y.-H.D.); mtdw2015@126.com (W.D.); schy1116@163.com (C.-Y.S.); wangk696@163.com (K.W.); akram@soe.ucsc.edu (M.-A.T.)

**Keywords:** microRNAs, myocardial infarction, myocardial cell death, cardiomyocyte regeneration, clinical application

## Abstract

MicroRNAs (miRNAs) are a class of small single-stranded and highly conserved non-coding RNAs, which are closely linked to cardiac disorders such as myocardial infarction (MI), cardiomyocyte hypertrophy, and heart failure. A growing number of studies have demonstrated that miRNAs determine the fate of the heart by regulating cardiac cell death and regeneration after MI. A deep understanding of the pathophysiology of miRNA dependent regulatory pathways in these processes is required. The role of miRNAs as diagnostic, prognostic, and therapeutic targets also needs to be explored in order to utilize them in clinical settings. This review summarizes the role of miRNAs in myocardial infarction and focuses mainly on their influence on cardiomyocyte regeneration and cell death including apoptosis, necrosis, and autophagy. In addition, the targets of pro- and anti-MI miRNAs are comparatively described. In particular, the possibilities of miRNA-based diagnostic and therapeutic strategies for myocardial infarction are discussed in this review.

## 1. Introduction

### 1.1. Introduction of Myocardial Infarction

Myocardial infarction (MI) is pathologically defined as myocardial cell death due to prolonged ischemia, which is the most severe manifestation of coronary artery disease (CAD). CAD causes over seven million deaths globally every year [1]. Every year, more than 700,000 people suffer from MI, which leads to 120,000 deaths every year in the United States alone. The economic impact of MI is substantial. The direct cost of hospitalization in the United States of America (USA) is at least US $450 billion and the loss of productive years of life is also tremendous [2].

The development of CAD can be chronic, by the erosion of endothelium and buildup of plaque and progressive narrowing of the coronary artery. However, a sudden rupture of plaque and formation of thrombus leads to acute MI, which is much more serious. Once the oxygen supply is occluded, the onset of MI is initiated as little as 20 min after and the complete myocardial cell necrosis happens in a few hours. Prolonged ischemia leads to the loss of heart contractility due to the poor proliferation capability of the myocardial cell. Therefore, the timely revascularization of the occluded artery is the key for MI therapy. Antithrombotic agents, percutaneous coronary intervention, and bypass surgery are usually applied to treat the patients [2,3]. However, these treatments only reduce the severity of CAD, rather than restoration of the contractility of the infarcted heart [3,4,5]. Therefore, novel therapeutic strategies to reduce the myocardial cell death and/or stimulate heart regeneration are highly desirable for the future.

### 1.2. Overview of miRNA

Although most of the genome is transcribed into RNAs, there is only 1–2% of human genome coding for proteins [6]. RNAs without protein coding potential, called non-coding RNAs, had been considered as “junk” product until recent studies confirmed that they are hugely involved in many biological processes. MicroRNAs (miRNAs) are defined as single-stranded non-coding RNAs with around 22 nucleotides. They were first discovered in *Caenorhabditis elegans* in 1993 [7,8] and now are the most extensively studied subgroup of non-coding RNA.

Most of miRNAs are generated by a canonical pathway. The miRNA coding genes are transcribed to miRNA precursors (pri-miRNAs) by either RNA polymerase II (pol II) or polymerase III (pol III). The pri-miRNAs contain a hairpin structure including the mature RNA sequence, a 5′ cap and a 3′ poly (A) tail. The 5′ cap and 3′ poly (A) are removed by a nuclear complex called microprocessor comprising Di-George syndrome critical region (DGCR) 8 and RNase III endonuclease Drosha. The resulting hairpin RNAs (pre-miRNAs) are then transported from the nucleus to the cytoplasm by Exportin-5 and RAN-GTP. In the cytoplasm the terminal loop of the hairpin structure is cleaved by a complex comprising of RNase III endonuclease Dicer, TAR RNA binding protein (TARBP) and protein kinase R (PKR) activator (PACT). The remaining RNA duplex interacts with Argonaute proteins (AGO) to form miRNA-induced silencing complex (miRISC), by which the passenger strand of miRNA is degraded and the mature miRNA retained [9].

In addition, some miRNAs are generated via non-canonical pathways, which may provide bona fide alternation to the canonical pathway. For example, small RNAs derived from endogenous short hairpin RNAs, tRNAs or tRNA like precursors, may bypass the Drosha-mediated process [10,11], during which Dicer processing is still required to remove the hairpin loop of these RNAs except for biogenesis of miR-451. In the case of miR-451, AGO catalytic activity directly cleaves pre-miR-451 in the middle of the 3′ strands of pre-miR-451 and further it is trimmed by PARN to generate a mature miR-451 [12,13].

### 1.3. Regulation of mRNA Translation and Decay by miRNA

**Figure 1:**
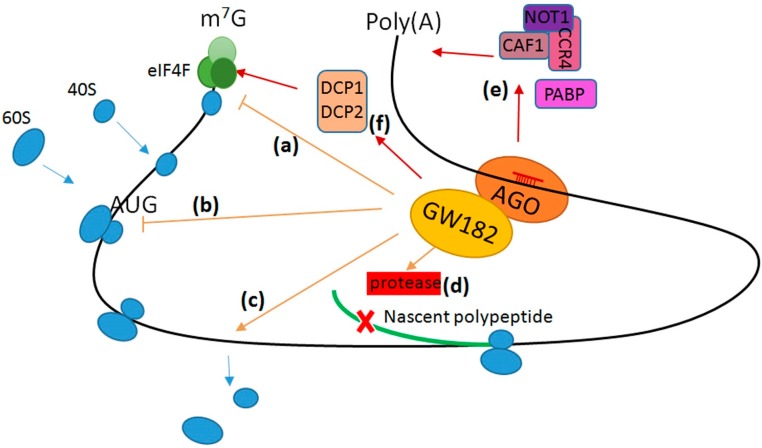
Schematic diagram of miRNA-mediated mRNA translational repression and decay. (a) miRNA-induced silencing coplex (miRISC) prevents the eIF4E-cap recognition and 40S ribosomal subunits recruitment; (b) 60S ribosomal subunit joining is prevented by miRISC; (c) Translation is inhibited by ribosome drop-off; (d) Translation is blocked by nascent polypeptide degradation; (e) miRISC interacts with Poly (A)-binding protein (PABP), CCR4-assocated factor (CAF1), negative on TATA-less (NOT1), and carbon catabolite repression 4 (CCR4), which mediate deadenylation, decapping, and mRNA decay; (f) miRISC interacts with decapping factor DCP1/2 for decapping and mRNA decay. Blue arrows represent the recruitment and disassociation of 40S/60S ribosomal subunits. Orange arrows represent the process fancilited by AGO/GW182. Orange T blocks represent the process prevented by AGO/GW182. Red cross (x), degradation of nascent polypeptide. Red arrows represent the interaction between AGO/GW182 and indicated factors and their function upon miRNA regulation.

MicroRNAs regulate protein synthesis usually by base-pairing with 3′ untranslated region (3′ UTR) of mRNAs, with some exceptions in which they interact with 5′ untranslated region (5′ UTR) of mRNA [14,15]. It is predicted that around 60% of total mammalian mRNAs could be regulated by miRNAs. The miRNAs achieve their function by binding with AGO proteins and other miRISC components like GW182 [14,15]. GW182 contains a GW repeats domain, glutamine (Q)-rich region, domain of unknown function (DUF), and a RNA recognition motif domain (RRM) and it usually works as a downstream of AGO. The C-terminal region including DUF and RRM is essential for translation repression [16,17]. miRNAs mediate translational repression either at the initiation step or the post-initiation steps [18]. At the initial stage of cap-dependent translation, the 40S ribosomal subunit binds to mRNA near 5′ cap and it scans the mRNA until it recognizes the start codon where the 60S ribosomal subunit joins the complex. miRNAs interact with the eIF4F complex and inhibit the recruitment of 40S ribosomal subunits to the 5′ cap. Additionally, miRNAs repress the initiation of translation by inhibiting the joining of the 60S ribosomal subunit [18] (Figure 1). A large body of evidence also supports the fact that miRNAs inhibit elongation and termination steps. During these steps miRNAs may either cause premature termination and subsequent ribosome drop-off, or recruit proteolytic enzymes that can degrade the nascent polypeptides, which leads to blockage of post-initiation processing [18,19,20,21].

In cytoplasm, mRNA is protected from degradation by the 5′ cap structure and a 3′ poly (A) tail. miRISC component GW182 recruits the carbon catabolite repression 4 (CCR4)- negative on TATA-less (NOT1) deadenylase complex to mRNA and facilitates AGO association with CCR4-assocated factor (CAF1). These factors are involved in the removal of poly (A) tail which subsequently results in mRNA decay [22,23,24]. GW182 also interacts with Poly (A)-binding protein (PABP), which mediates both translation initiation and mRNA deadenylation [24]. In addition, GW182 mediates 5′ decapping via interaction with decapping factor DCP1 and DCP2 [25]. All together miRNAs mediate mRNA decay via both the deadenylation and decapping mechanism [18].

## 2. Specific Function of MicroRNAs in Cardiac Cell Death

**Figure 2:**
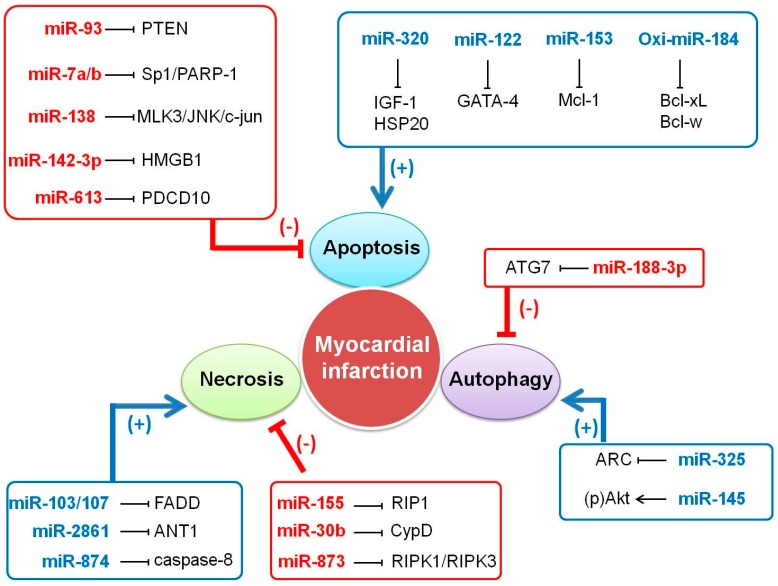
Overview of the role of miRNAs-regulated myocardial cell death in MI. See text for detailed explanations. Blue arrows and plus sign indicate that the final effect is the activation of cell death. T bars and minus sign indicates that the final effect is the inhibition of cell death.

Cardiac cell death plays a critical role in the pathogenesis of myocardial infarction, due to the terminal differentiation and loss of regenerative ability of cardiomyocytes. Myocardial infarction usually involves three main types of cell death process including apoptosis, necrosis, and autophagy [26,27,28]. Cardiac cell death processes are regulated by a variety of molecules, among which miRNAs have shown outstanding regulatory functions. Recently, a large body of research has emphasized the importance of miRNAs in regulating apoptosis, necroptosis, and autophagy in cardiomyocytes, which play a decisive role in myocardial infarction (Figure 2). The latest findings on the specific function of miRNAs in cardiac cell death are discussed in the following sections.

### 2.1. Apoptosis

Apoptosis is a type of programmed cell death executed through the extrinsic pathway through death receptors [29] and/or the intrinsic pathway mainly by mitochondria [30,31]. The signal transduction of apoptosis is mediated by many pro-apoptosis and/or anti-apoptosis factors including caspase family [31], Bcl-2 family, [32] cytochrome c [33], and p53 [34].

miRNAs regulate apoptosis usually through targeting pro-apoptotic factors-related pathways. A great variety of apoptosis-related miRNAs or miRNA families have been identified. Much attention has been paid to the let-7 family of miRNAs [35], miR-34 family, [36,37] miR-21 family [38,39], miR-30 family [40], miR-125b [41], and miR-138 [42] in the field of cardiac diseases due to their impact on cardiomyocyte apoptosis. Besides, the research on the link between miRNA and apoptosis regulatory network is expanding and more pro- and anti-apoptotic miRNAs have been recently identified. In an experimental model, overexpression of miR-93 inhibits cardiomyocyte apoptosis by targeting phosphatase and tensin homolog (PTEN) in mice with ischemia/reperfusion (I/R) injury [43]. In adult cardiomyocytes (H9C2), hypoxia inducible apoptosis can be inhibited by miR-7a/b-Sp1/PARP-1 pathways [44] while miR-138 exhibits a protective effect against hypoxia-induced apoptosis via MLK3/JNK/c-jun pathway in H9C2 cell lines [42]. Similarly, miR-142-3p targets the high mobility group box 1 (HMGB1) [45] and miR-613 targets PDCD10 [46] and inhibits apoptotic cell death in cardiomyocytes. In contrast, miR-320 promotes apoptosis by targeting insulin-like growth factor-1 (IGF-1) which inhibits cardiomyocyte apoptosis by down-regulating p-ASK, p-JNK, p-p38, Bax and Caspase-3 levels, and upregulating Bcl-2 levels. Inhibition of miR-320 up-regulates the level of IGF1 mRNA [47]. Likewise, the knockdown of miR-122, an apoptosis-related microRNA, attenuates hypoxia/reoxygenation-induced myocardial cell apoptosis by upregulating GATA-4 [48]. In oxidative stress condition, the expression of miR‑153 is significantly increased, and inhibition of endogenous miR‑153 can block cardiomyocyte apoptosis [49].

In particular, an inspirational study demonstrated that oxidative modification of miRNAs is implicated in cardiomyocyte apoptosis. Wang et al. identified that several miRNAs are oxidatively modified by various oxidant systems, among which miR-184, miR-204-3p, and miR-139-3p are highly oxidized under oxidative stress condition. They observed that this oxidation enables miRNAs to negatively regulate gene expression through mismatching, whereas the native miRNAs could not. For instance, the oxi-miR-184 mismatches with the 3′ UTRs of anti-apoptotic genes Bcl-xL and Bcl-w and leads to reduction in their levels, which subsequently leads to apoptotic cell death of cardiomyocytes. The oxidized miR-204-3p inhibits pancreatic and duodenal homeobox 1C-terminal inhibiting factor (Pcif1) and oxi-miR-139-3p substantially induces a reduction in the luciferase activity and protein levels of RNA (guanine-7-) methyltransferase (RNMT), but not by native miR-204-3p or miR-139-3p. These findings enrich miRNAs-based pathways in regulating apoptosis [50].

Thus, miRNAs function as angels or devils in apoptosis regulatory network. It is expected that more miRNAs and their targets in cardiomyocyte apoptosis program could be unveiled in the future.

### 2.2. Necroptosis

For a long time, Necrosis was considered as a passive and accidental form of cell death. In recent years, a growing number of studies demonstrated that necrosis is regulated by multiple regulators and hence termed as “programmed necrosis” or “necroptosis”. Necroptosis is usually initiated by tumor necrosis factor (TNF)-α and TNF-α receptor (TNFR1), and then mediated by receptor-interacting serine/threonine-protein kinase (RIPK) 1 and 3 pathways [51].

Recent research demonstrated that miRNAs participate in necrotic process in cardiac disorders. In H9C2 cells, miR-103/107 levels are elevated in H_2_O_2_-induced necrosis and knockdown of endogenous miR-103/107 antagonizes necrotic cell death. The molecular study revealed that miR-103/107 provokes cardiomyocyte necrosis through targeting Fas-associated protein with death domain (FADD) [27]. Cyclophilin D promotes necrosis in myocardial infarction, which is suppressed by miR-30b through inhibition of its translation. Cardiac-specific enhancement of miR-30b expression in transgenic mice exhibits reduced necrosis and myocardial infarct size following ischemia/reperfusion injury [52]. Similarly, overexpression of miR-155 attenuates necrotic cell death via targeting RIP1 in cardiomyocyte progenitor cells [53]. miR-2861 and adenine nucleotide translocase 1 (ANT1) constitute an axis to promote necrosis in the heart. Forced expression of miR-2861 triggers H_2_O_2_-induced necrotic cell death through targeting ANT1, while knockdown of miR-2861 attenuates necrosis [54]. Another necrosis promoter miR-874 was demonstrated to play a key role in oxidant induced necrosis pathways by comprising Foxo3a and caspase-8 [55]. miR-873 inhibits RIPK1/RIPK3-mediated necrotic cell death in cardiomyocytes [56]. Although miRNAs have been demonstrated to be associated with necroptosis, the underlying mechanism remains unclear. Therefore, the influence of miRNAs in the necroptosis process need to be further clarified in future studies.

### 2.3. Autophagy

Autophagy is characterized by an evolutionarily conserved process of lysosome-dependent degradation of cytoplasm components and damaged organelles such as endoplasmic reticulum, peroxisomes, and mitochondria. Generally, autophagy is an eliminating process of intracellular pathogens. Autophagy proceeds in several successive stages including induction, nucleation, expansion, and maturation [57]. In the heart, autophagy is required for the maintenance of cardiomyocytes homeostasis, while abnormal autophagy leads to the development of cardiac disorders [58,59].

Autophagy is widely implicated in cardiac pathology and it is regulated by microRNAs [28,59,60,61,62,63,64]. For example, Wang et al. demonstrated that miR-188-3p functions as an autophagy inhibitor in myocardial infarction. The level of miR-188-3p is reduced both in cardiomyocytes treated with anxia/reoxygenation (A/R) and mice upon myocardial infarction, but enforced expression of miR-188-3p can alleviate autophagic cell death in cardiomyocytes induced by A/R. In the mouse model of myocardial infarction, overexpression of miR-188-3p attenuates autophagy and infarct size through targeting autophagy mediator ATG7. These findings suggest that miR-188-3p plays a cardioprotective role in myocardial infarction by inhibiting autophagy [59]. Another research work revealed that miR-145 repairs infarcted myocardium by accelerating cardiomyocyte autophagy in a rabbit model of myocardial infarction [28]. miR-19a-3p/19b-3p suppresses autophagy of human cardiac fibroblasts through targeting transforming growth factor (TGF)-β receptor II mRNA. In the circulation of heart failure patients, the level of miR-19a-3p/19b-3p is very low. The miR-19a-3p/19b-3p overexpression promotes collagen 1α2 and fibronectin synthesis in human cardiac fibroblasts, which can be interrupted by autophagy inhibitor. This enlightens the influence of miR-19a-3p/19b-3p on the autophagic process [61]. Bim is another downstream target of miR-19a-regulated-autophagy pathway [62]. miRNAs-regulated-autophagy plays a critical role in pathological cardiomyocyte hypertrophy. Ucar et al. revealed that the miR-212/132 family regulates cardiac hypertrophy and autophagy in cardiomyocytes. miR-212/132 knockout mice exhibit a cardioprotective effect against pressure-overload-induced heart failure, while cardiomyocyte-specific overexpression of the miR-212/132 family leads to pathological cardiac hypertrophy. Further studies identified that transcription factor FoxO3 is a target of miR-212/132 in autophagic signal pathway [64]. Taken together, miRNAs are closely linked to autophagy-related cardiac pathology, however, whether mRNAs depdendent autophagy provokes or inhibits cardiac disorders seems to be confused, and hence this necessitates further elucidation of the underlying molecular mechanism.

In summary, the broad involvement of miRNAs in cardiac cell death has been exhibited. A growing number of miRNAs as well as their targets have been identified in apoptosis, necroptosis, and autophagy regulation. Nevertheless, more regulatory pathways and the link between different pathways of miRNA regulating cardiac cell death need to be clarified urgently.

## 3. How Do MicroRNAs Regulate Myocardial Infarction?

The significance of miRNAs in regulating myocardial infarction has been well emphasized by multiple studies. Several miRNAs are downregulated/upregulated depending on the type of myocardial injury. The significant changes in their expression pattern upon myocardial infarction highlights their contribution in regulation of pathogenesis of MI. The miR-15 family including six closely related-miRNAs are upregulated both in the mouse and pig models of myocardial infarction [65]. The level of miR-34 family members (miR-34a, -34b, and -34c) are significantly increased in mice subjected to MI [66]. miR-499 is downregulated in the mouse model of MI [67]. miR-320 expression is significantly decreased in the heart following I/R injury [68]. miR-24 expression is depressed in heart tissue after myocardial infarction [69]. Additionally, the levels of expression of miR-1 [70], miR-16 [71], miR-21 [39], miR-92a [72], miR-195 [73], miR-208 [74], miR-375 [75], miR-494 [76], miR-103/107, miR-325, and miR-874 are significantly upregulated in heart tissue myocardial infarction, while the levels of tissue miR-133a/b [74], miR-214 [77], miR-873 [56], miR-2861 [54], miR-30b [52], miR-188-3p [59], and miR-145 [28] are decreased upon MI.

MicroRNAs play a critical role in myocardial infarction through regulating apoptotic, necrotic, and autophagic cell death. Among the aberrantly expressed miRNAs in MI, several typical miRNAs are chosen here to illustrate how miRNAs regulate myocardial infarction. miR-494 can protect hearts against I/R-induced injury by targeting both pro- and anti-apoptotic proteins. miR-494 transgenic mice exhibit improved recovery of contractile performance accompanied by apoptosis inhibition during the reperfusion period [76]. Down-regulation of miR-320 suppresses cardiomyocyte apoptosis and improves heart function against myocardial infarction [47]. Inhibition of miR-103/107 elevation reduces infarct size and improves cardiac function after ischemia/ reperfusion by alleviating cardiac cell programmed necrosis [27]. miR-874 displays its potential positive influence on myocardial necrosis by suppressing Foxo3a, which is implicated in the development of myocardial infarction [55]. Similarly, other microRNAs such as miR-873 and miR-2861 [54] can reduce myocardial infarct size by attenuating ischemia/reperfusion induced programmed necrotic cell death [56]. Cardiac-specific expression of miR-30b in transgenic mice exhibits reduced necrosis and myocardial infarct size in ischemia/reperfusion [52]. miR-188-3p suppresses autophagy and myocardial infarction by targeting ATG7 [59], While myocardial infarction is promoted by miR-325 regulated autophagic cell death [60]. miR-145 repairs infracted myocardium by accelerating cardiomyocyte autophagy [28] Collectively, these findings depict the complicated network of miRNAs in myocardial infarction.

## 4. MicroRNAs Regulate Cardiomyocyte Regeneration in Myocardial Infarction

The mammalian heart has only a limited regenerative capacity. The hearts of neonatal animals have a remarkable capability for cardiac repair and regeneration following injury caused by myocardial infarction [78]. However, the regenerative capacity largely declines within seven days after birth and remains very low in the adult heart [78]. Thus, the activation of endogenous heart regeneration and the triggering of cardiomyocytes renewal could provide new clues for the therapy to treat myocardial infarction. Different approaches have been proposed to regenerate new cardiomyocytes: (1) to promote resident cardiomyocytes proliferation by inducing them to re-enter the cell cycle; (2) to activate endogenous stem cells or progenitors such as cardiac stem cells (CSCs) differentiation; (3) to stimulate endogenous regeneration through direct reprogramming from cardiac fibroblasts into cardiomyocytes [26]. It has been demonstrated that miRNAs are critical regulators of these processes and exhibit as potential new therapeutic targets for MI.

**Figure 3:**
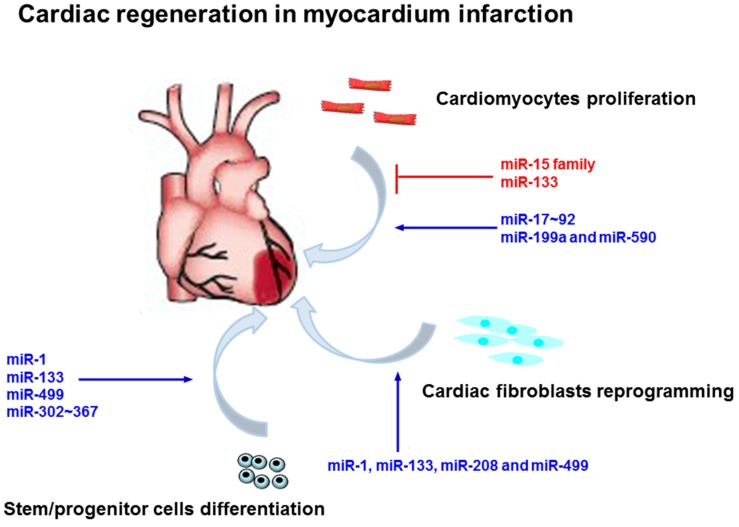
Summary of miRNAs functioning to regulate cardiac regeneration in MI. Cardiomyocytes proliferation, cardiac fibroblasts reprogramming and stem/progenitor cells differentiation mediated by miRNAs are shown in the figure. See text for detailed explanations. Both blue and grey arrows represent the promotion effect. T bars represent the inhibition effect.

Like many other organ development processes, mammalian heart development and homeostasis is tightly regulated by miRNAs (Figure 3). As cardiomyocytes stop proliferating shortly after birth in mice, the miRNA expression profile in this period shows a series of changes related to the progressive decrease of cardiomyocytes proliferation capacity. The miR-17~92 cluster including miR-17, miR-18a, miR-19a, miR-19b, miR-20a, and miR-92a, has been demonstrated to be associated with cardiomyocytes proliferation [79]. Overexpression of this cluster using cardiomyocyte-specific knock-in mice substantially raises the total number of cardiomyocytes, which increases the wall thickness and left ventricle dimension, while conditional knockout of this cluster in the heart notably reduces cardiomyocyte proliferation and heart weight at birth [79]. However, overexpression of miR-17~92 in the adult heart alleviates MI injury responses [79]. Recently, miR-548c-3p, miR-509-3p, and miR-23b-3p were shown to induce significant proliferation in adult cardiomyocytes through translational inhibition of Meis1 and thereby regulate cell-cycle progression [80]. In contrast, miR-590 and miR-199a encourage cell cycle re-entry of adult cardiomyocytes ex vivo and promote cardiomyocyte proliferation in neonatal mice. Interestingly, a single administration of synthetic miR-590/miR-199a-lipid formulations was sufficient to stimulate cardiac repair and restoration of cardiac function [81,82]. In mice suffering MI, miR-590 and miR-199a completely improved cardiac function through stimulation of cardiac regenerative processes [81]. In addition, the enhancement of miRNA-204 expression promotes cardiomyocyte proliferation in both neonatal and adult cardiomyocytes by targeting Jarid2. Transgenic mice with the cardiac-specific overexpression of miRNA-204 exhibited excessive cardiomyocyte proliferation throughout the embryonic and adult stages which led to a pronounced increase in ventricular mass [83].

Many other miRNAs have been demonstrated to inhibit cardiomyocytes proliferation. The miR-15 family (including miR-15a, miR-15b, miR-16, miR-195, and miR-497) is a potential inhibitor of cardiomyocytes proliferation [84]. Inhibition of miR-15b and miR-16 extends the period of cardiomyocyte proliferation after birth in mouse [84]. However, the overexpression of miR-195 in transgenic mice exhibits a marked reduction in the number of cells undergoing mitosis and increased proportion of multinucleated cardiomyocytes at the early postnatal period [85]. The heart from these mice displayed cardiac malformations including ventricular septal defect [85]. In addition, miR-133a is known as an anti-proliferation factor in cardiomyocytes. Genetic deletion of miR-133a leads to 50% embryonically lethality rate in mice, and surviving mice acquire cardiomyopathy as well as heart failure due to aberrant proliferation of cardiomyocytes [86]. In model system like zebrafish, the depletion of miR-133 induces cardiomyocytes proliferation and promotes quick recovery of the injured heart, while overexpression of miR-133 inhibits this process [87].

Stimulating proliferation and differentiation of endogenous stem cells or progenitors like cardiac progenitor cells (CPCs) is another alternative strategy to compensate cardiomyocytes loss. miRNAs are linked to such processes and their actions promote cardiomyocytes regeneration [88]. miR-1 and miR-133 family are rapidly upregulated during in vitro differentiation of adult CPCs [89]. Overexpression of miR-1 family enhances the cardiac differentiation of CPCs, whereas overexpression of miR-133 family does not modulate this process but protects CPCs against apoptosis [89]. Likewise, forced expression of miR-499 in human CPCs can promote the differentiation to CMs in vitro [90]. miR-300 is a poorly characterized microRNA in the Dlk1-Dio3 microRNA cluster, which positively regulates Bmi1 in CPCs. Forced expression of miR-300 in CPCs promoted an improved stemness signature but significantly reduced key cardiac transcription factors, including Nkx2.5 and Tbx5 [91]. In the early development of the heart, some miRNAs were shown to maintain tissue-specific progenitors [92]. miR-302~367 clusters (including miR-302a, miR-302b, miR-302c, miR-302d and miR-367) encourage embryonic cardiomyocytes proliferation by targeting CPCs [93]. miR-302~367 gain of function leads to cardiomegaly in fetal and juvenile hearts with a more undifferentiated cardiomyocytes phenotype, similar to mature hearts. The infarcted mouse hearts treated systemically with miR-302~367 exhibit decreased cardiac fibrosis and improved heart function [93]. In addition, forced expression of miR-499 in human CPCs could promote the differentiation to CMs in vitro [90].

Cardiac fibroblasts comprise over 50% of the whole cells in the heart [94]. Fibroblasts can be successfully reprogrammed into cardiomyocytes with a combination of the transcription factor, which reduces infarct size of hearts and improves cardiac function in mice upon MI [95]. Several studies demonstrate the involvement of miRNAs in mediating fibroblasts reprogramming. For instance, miR-1, miR-133, miR-208 together with miR-499 (named miR combo) are sufficient to induce efficient reprogramming of cardiac fibroblasts into cardiomyocytes both in vitro and in vivo [96]. This combination promoted a progressive improvement in cardiac function as well as reduced fibrosis over a 3-month period after MI [97].

## 5. MicroRNAs as Biomarkers and Clinical Therapeutic Targets in Myocardial Infarction

An ideal biomarker usually fulfills three criteria: good accessibility, predictability of detection, and robust reliability [98]. Currently more sensitive and specific biomarkers are required in clinical practice. Although serum troponin is now being used as a diagnostic marker for MI, its delayed release time often leads to low sensitivity. Recently, the identification of alterations of miRNAs as a cardiac response to ischemia has motivated investigations for new perspectives of clinical research. Circulating miRNAs in the blood have recently emerged as potential biomarkers for the diagnosis or prognosis of MI due to their stability and specificity in plasma. A large body of studies explored the fact that miRNAs are leaked from the heart into the circulation after myocardial injury [99,100], during which their expression is elevated and dynamic [101,102]. Circulating miRNAs are stable and can be easily quantified by real-time PCR assay. Among these abundant miRNAs in the heart, four cardiac-enriched miRNAs (miR-208, miR-499, miR-1, and miR-133) are consistently found to be increased in the plasma of acute myocardial infarction patients [74,103,104]. For example, Wang et al. measured circulating levels of miRNAs in patients with AMI as well as in healthy subjects. They observed a significant up-regulation of miR-1, miR-133, and miR-499 in the circulation of AMI patients [102]. Interestingly, miR-208a is highly detectable in 91% of AMI patients but not in healthy controls. In addition, side-to-side comparisons of receiver operating characteristic (ROC) curves reveal that miR-208a has advantages over troponin in the early phase of MI. Other small-scale studies also confirmed that these four miRNAs are linked to MI in cultured cardiomyocytes as well as in patients [103,105,106]. Recently, the efficiency of circulating miR-92a and miR-181a as potential novel biomarkers for diagnosis of AMI patients was reported [107,108]. Based on these results, further large-scale studies on patient populations are required to confirm their utility as biomarkers. Oerlemans et al. determined the potential value of circulating miR-1, miR-21, miR-146a, miR-208a, and miR-499 in a cohort of 332 suspected acute coronary syndrome (ACS) patients, and found that the combination of miR-1, miR-21, and miR-499 could have a higher diagnostic value than hs-troponin T [109]. In a study with a total of 424 patients presenting with ACS, miR-208b and miR-499 showed a higher expression level in MI patients compared with non-MI patients, and they were well correlated with cardiac troponins. However, their diagnostic value is not superior to cardiac troponins. [104]. Another study performed with 1155 patients with acute chest pain also confirmed that none of the tested miRNAs outperformed cTnT [110]. These identified circulating miRNAs as diagnostic biomarkers for MI are listed in Table 1. Although the potential value of miRNAs as biomarkers has been established in small-scale studies, it is difficult to validate them in large cohorts of patients with MI. In addition, the methods of miRNAs detection need to be optimized. The standardized assays for the detection of miRNAs in patients may reduce the inconsistency and miRNAs may become potential biomarkers for diagnosis of MI patients. More prospective studies are underway to assess the diagnostic value of miRNAs as biomarkers.

On the other hand, predicting the outcome after MI can offer information about cardiac dysfunction [111]. However, there are still no appropriate prognostic biomarkers recommended for clinical practice, although a number of candidate prognostic biomarker of MI have been identified, such as neutrophil gelatinase-associated lipocalin (NGAL), brain natriuretic peptide (BNP), and soluble ST2 (sST2) [111]. Among them, BNP is currently considered to be an optimal prognostic biomarker for MI, however, it lacks public acceptance due to its long half-life and fluctuant levels in plasma upon MI [112]. The sensitivity and specificity of circulating miRNAs have an attractive prognostic value in response to MI, and relevant research is being designed and carried out.

The discovery of miRNAs involvement in the cardiac response to ischemia has captured a lot of attention in exploring the therapeutic regulation of miRNA. So far, several strategies have been undertaken to enhance the levels of miRNAs with salutary functions or to knock down the expression of pathogenic miRNAs in vivo. miRNA mimics are synthesized to up-regulate the expression of interest miRNA with double-stranded structure, in which one “guide” strand is identical to the mature miRNA and the other “passenger” strand is complementary to its guide strand of miRNA with position-specific chemical modifications to ensure location into the RISC [113]. On the contrary, chemical modifications such as 2′-*O*-methyl-modified oligonucleotides with perfect complementarity to target miRNAs (antagomiR), are the most often used techniques in anti-miRNAs strategy. Besides, 2′-*O*-methoxyethyl (MOE)-modified oligonucleotides and locked nucleic acid (LNA)-modified oligonucleotides have also been applied to inhibit miRNA activity. Numerous studies support this strategy by showing the delivery of antagomiR in animals effectively reduced target miRNA levels for a long time with low toxicity [114]. For example, Care et al. observed inhibition of miR-133 using an antagomiR blocked cardiac hypertrophy in mice [115]. Systemic delivery of LNA-modified oligonucleotides (antimiR-208a) during heart failure effectively improved cardiac function and survival in rats [116]. Although tremendous progress has been made in miRNAs-based therapeuticsfor MI, the most important challenges such as the stability in specific organs or cell types remain to be solved before their clinical application.

## 6. Conclusions and Perspectives

A lot of attention has been paid to delineate the association between miRNAs and myocardial infarction over a long time span. Accumulating evidence reveals that miRNAs function as pro- or anti-MI factors through their influence on myocardial cell death and cardiomyocyte regeneration pathways. So far, significant progress has been made to unveil the miRNAs-regulated signaling pathways of myocardial infarction, which has improved our understanding of heart pathogenesis. In fact, the expression levels of many miRNAs are highly altered in the plasma of MI patients, which emphasizes the potential of miRNAs in the diagnostics and therapeutics of MI. Notably, the miRNA-based therapeutic approach has had a remarkable outcome in a variety of experimental models of MI in animals. These advancements appear as a light in the darkness and provide real hope in the fight against myocardial infarction.

## Figures and Tables

**Table 1:** Circulating miRNAs as diagnostic biomarkers for MI.

miRNAs	Study Design	Source	Reference
miR-221-3p	27 AMI ^a^ patients; 16 control subjects	Plasma	[117]
miR-21	17 AMI patients; 10 control subjects	Plasma	[118]
miR-423			
miR-19b-3p	18 AMI patients; 20 control subjects	Plasma	[119]
miR-134-5p			
miR-186-5p			
miR-1	93 AMI patients; 66 control subjects	Plasma	[120]
miR-133a/b	33 AMI patients; 17 control subjects	Plasma	[121]
miR-499			
miR-122			
miR-375			
miR-208a	33 AMI patients; 30 control subjects	Plasma	[102]
miR-328			
miR-320a	224 AMI patients; 931 non-AMI patients	Plasma	[110]
miR-146a	106 ACS ^b^ patients; 226 non-ACS patients	Serum	[109]

^a^ AMI, acute myocardial infarction; ^b^ ACS, acute coronary syndrome.

## Abbreviations

3′ UTR	3′ untranslated region
5′ UTR	5′ untranslated region
ACS	Acute coronary syndrome
AGO	Argonaute proteins
AMI	Acute myocardial infarction
ANT1	Adenine nucleotide translocase 1
A/R	anxia/reoxygenation
BNP	Brain natriuretic peptide
CAD	Coronary artery disease
CAF1	CCR4-assocated factor
CCR4	Carbon catabolite repression 4
CPCs	Cardiac progenitor cells
CSCs	Cardiac stem cells
DGCR	Di-George syndrome critical region
DUF	Domain of unknown function
FADD	Fas-associated protein with death domain
HMGB1	High mobility group box 1
IGF-1	Insulin-like growth factor-1
I/R	Ischemia/Reperfusion
LNA	Locked nucleic acid
MI	Myocardial infarction
miRNAs	microRNAs
miRISC	miRNA-induced silencing complex
MOE	2’-O-methoxyethyl
NOT1	Negative on TATA-less
NGAL	neutrophil gelatinase-associated lipocalin
PABP	Poly (A)-binding protein
PACT	protein kinase R activator
Pcif1	pancreatic and duodenal homeobox 1C-terminal inhibiting factor
PKR	protein kinase R
pol II	Polymerase II
pol III	Polymerase III
pre-miRNAs	Hairpin miRNAs
pri-miRNAs	Precursors of miRNAs
PTEN	Phosphatase and tensin homolog
RIPK	Receptor-interacting serine/threonine- protein kinase
RNMT	RNA (guanine-7-) methyltransferase
ROC	Receiver operating characteristic
RRM	Recognition motif domain
sST2	soluble ST2
TARBP	TAR RNA binding protein
TGF	transforming growth factor
TNF	Tumor necrosis factor
TNFR	Tumor necrosis factor receptor
